# Effect of autogenous crack self-healing on mechanical strength recovery of cement mortar under various environmental exposure

**DOI:** 10.1038/s41598-021-86596-2

**Published:** 2021-03-31

**Authors:** A. R. Suleiman, M. L. Nehdi

**Affiliations:** Department of Civil and Environmental Engineering, Western University, London, ON N6A 5B9 Canada

**Keywords:** Engineering, Materials science

## Abstract

While research on self-healing of cement-based materials has recently gained considerable attention and made sizable progress, there is still ongoing debate and controversy regarding the effect of crack closing induced by autogenous self-healing on mechanical strength recovery. Despite that several techniques have been used to capture and quantify the self-healing of surface cracks, the resulting effect on mechanical strength has not, to date, been explored and quantified in a rigorous and systematic manner. Therefore, in this study, a broad array of multi-scale techniques including non-destructive shear wave velocity, high-resolution X-ray computed tomography (µCT), and 3D image analysis was deployed to examine the effects of autogenous crack self-healing on the mechanical strength recovery in various mortar specimens. The influence of microstructural changes induced by additives such as swelling compounds, silica-based additions, and carbonating minerals on strength recovery under diverse environmental exposures was further explored. The results capture the relationship between the crack closing mechanism imparted by self-healing and mechanical strength recovery, therefore elucidating the discrepancies in mechanical strength recovery results reported in the open literature.

## Introduction

Concrete is a brittle material with high cracking susceptibility. Cracking can result from a variety of causes that include plastic, autogenous and drying shrinkage, thermal stress, mechanical loading, differential settlement, and various other damage mechanisms^1–3^. This cracking behaviour, which is nearly inevitable, can jeopardize the mechanical strength and durability performance of concrete civil infrastructure. Therefore, concrete structures require routine crack monitoring and repair, which can be costly and time-consuming, and especially challenging in large-scale infrastructure like dams, bridges, tunnels, and difficult to access areas^4–6^.

According to the 2019 Canadian Infrastructure Report Card, Canada’s public infrastructure is at risk, with a colossal backlog of aging structures requiring immediate attention. Likewise, in the USA, the most recent infrastructure report card shows that more than 55,000 of the nation’s bridges are structurally deficient and need urgent repair. Similar challenges have been reported elsewhere^7^. Hence, infrastructure deterioration has become a notorious global problem draining massive financial resources.

In the last decade, several studies have explored the potential of developing self-repairing concrete having crack self-healing ability. This could greatly reduce maintenance and repair costs, preserve infrastructure functionality, decrease greenhouse gas emissions, and produce more sustainable structures. Accordingly, different techniques have emerged to assess the efficiency of self-healing in concrete (e.g.^8–27^). For instance, Azarsa et al.^24^ investigated the effect of crystalline additives on the self-healing of concrete based on water permeability tests. Cylindrical specimens were first cracked and then exposed to a constant water-head from one end. They found that control samples exhibited the lowest self-healing rate, while specimens incorporating crystalline admixtures achieved around 90% self-healing. Liu et al.^28^ used a simulated marine environment to study the effects of seawater ions and drying-wetting cycles on autogenous self-healing of cracks in cement paste. In their study, a stereomicroscope was used to quantify the crack healing ratio. It was found that cracks in cement paste specimens subjected to a simulated marine environment achieved better self-healing than that of specimens immersed in tap water. Rong et al.^29^ studied the influence of bacterial concentration in cement mortars using image processing techniques and found that the healing efficiency increased as the bacterial concentration increased.

Several studies have reported precipitation or crystallisation of CaCO_3_ as the main healing mechanism of surface cracks^13,16,19–21,27,30–33^. For instance, Zhu et al.^33^ investigated the self-healing of engineered cementitious composites exposed to freezing and thawing cycles. They found that, for both exposure conditions considered, the formation of CaCO_3_ sealed the cracks. Tomczak and Jakubowski^30^ explored crack healing in high-cement content and low w/c ratio composites. They also found that CaCO_3_ was the primary healing compound. Similar results were reported for concrete using bacterial spore induced self-healing^19^.

An extensive survey of pertinent studies in the open literature shows that self-healing in concrete has been primarily studied from a durability perspective, particularly considering crack closure behavior, chloride ions diffusion, and liquid and gas tightness recovery. Mechanical strength recovery of cement-based composites, such as engineered cementitious composites (ECCs), has also been extensively investigated and reported^33–39^. For example, Şahmaran et al.^37^ studied the intrinsic self-healing ability of engineered cementitious composites under mechanical loading. Their results showed that ECC specimens could recover up to 85% of their initial resonant frequency depending on the type of supplementary cementitious material used. Zhu et al.^33^ explored the self-healing of ECCs exposed to two different cyclic freezing/thawing regimes and found that the degree of self-healing under fresh water freezing/thawing cycles was higher than that in de-icing saltwater freezing/thawing cycles. Qian et al.^38^ reported that ECC specimens with higher cementitious materials content can exhibit better mechanical strength recovery.

However, to date, the effect of self-healing induced crack closing in concrete on its mechanical strength recovery has not been duly studied. According to Li et al.^40^, the self-healing mechanism is a localized phenomenon on a micro-scale level that can hardly be captured through conventional destructive testing methods. It rather requires non-destructive testing techniques that are sufficiently sensitive to detect mechanical strength recovery on a micro-scale level. The shear wave velocity technique (SWV) is a non-destructive technique that has been employed in several fields including geotechnical, structural, and medical science applications. For example, Mehta and Antich^41^ assessed the biomechanical competence of bone using SWV. Cortez et al.^42^ used SWV measurements to detect musculoskeletal abnormalities. Liu et al.^43^, Birgül^44^, An et al.^45^, Soliman et al.^46^, and Zhu et al.^47^ evaluated different properties of concrete using SWV testing. Several other studies have used SWV to investigate the properties of different soils and rocks (e.g.,^48–52^).

Previous studies have investigated various properties of concrete using ultrasonic measurements. For instance, Liu et al.^43^ used ultrasonic shear waves to investigate the setting and hardening progression in cement-based specimens. Their results indicated that shear wave velocity can detect the setting and hardening process of cement-based materials. Similarly, Soliman et al.^46^ reported that early-age properties of concrete can be monitored using the shear-wave velocity technique. Naji et al.^53^ evaluated the potential segregation of self-consolidating concrete through measuring changes in shear waves using a piezoelectric ring actuator. They found that static stability of SCC concrete can be assessed using such a technique. An et al.^45^ posited that shear wave velocity can be applied to characterize the compressive strength development of concrete under various curing regimes. The encouraging results outlined above motivated the use of shear wave velocity measurements in the present study to explore the effects of crack self-healing on mechanical strength recovery in cement mortar.

## Research significance

Research on the self-healing of cement-based materials has received a great deal of attention over the last decade. However, the effect of autogenous crack closing due to self-healing on mechanical strength recovery of cement-based materials has not been duly investigated. Therefore, several techniques were used in the current study to explore the influence of autogenous crack self-healing on mechanical strength recovery of cement-based materials under diverse environmental exposures. For the first time, a sensitive non-destructive shear wave velocity test was used along with X-ray micro-computed tomography (Xray μCT) coupled with 3-D image processing and analysis to elucidate the relationship between mechanical strength recovery and autogenous crack self-healing in cement-based materials. Shear wave velocity testing allowed to measure mechanical strength recovery in mortar specimens incorporating various additives under diverse environmental exposure. Xray μCT and 3-D image analysis provided visualization of the extend of self-healing and quantifying, for the first time, the entire crack volume changes due to autogenous self-healing. Scanning electron microscopy coupled with X-ray spectroscopy shed more light on the composition and morphology of the healing products formed within the cracks. Moreover, the evolution of microstructure densification of the uncracked portion of the tested specimens versus time was quantified using mercury intrusion porosimetry. The combined results from these techniques provide a robust assessment of the effect of autogenous self-healing induced crack closure on mechanical strength recovery of cement-based materials.

## Laboratory testing

### Materials, mixture proportions, and testing procedures

**Figure 1:**
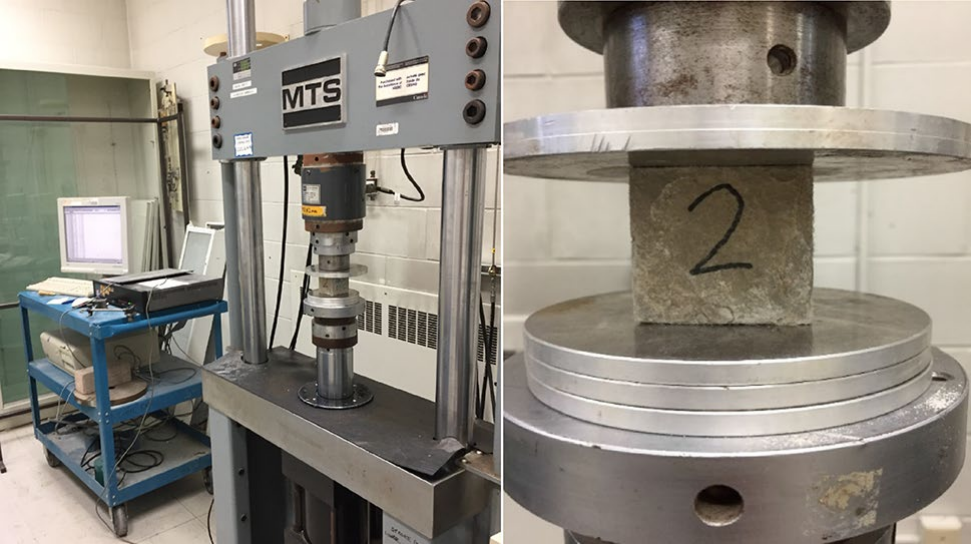
Cracking of mortar specimens using MTS machine.

**Figure 2:**
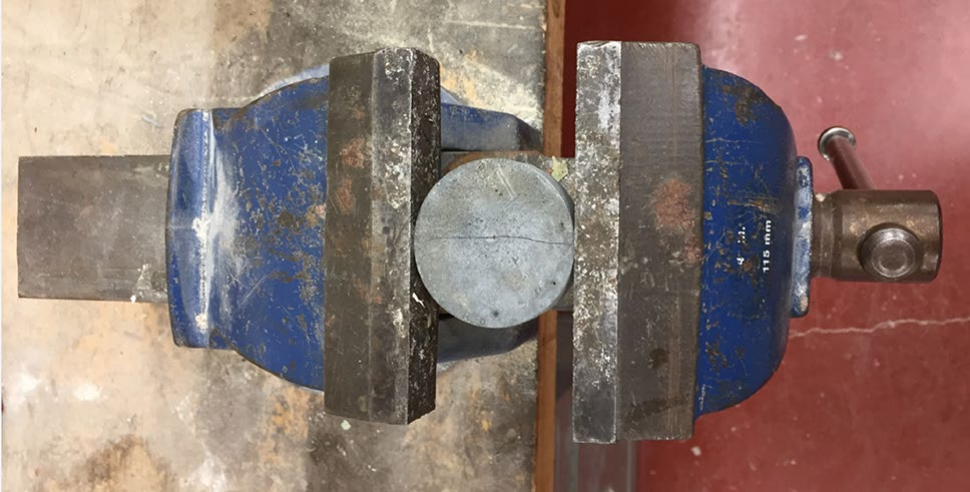
Cracking of mortar specimens using screw jack.

The materials used in this study include portland cement (CSA A3001, Type GU), polyvinyl alcohol (PVA) fiber, bentonite (BN), fine calcium carbonate powder (CC), high reactivity metakaolin (MK), and fly ash (FA). The physical and chemical properties of these materials are summarized in Tables 1 and 2. The mixture proportions investigated are detailed in Table 3. For all mixtures, PVA fiber at 1% by volume fraction was added. All dry ingredients were mixed using a Hobart mixer. Water was added at a water-to-binder materials mass ratio of 0.35. To control the workability and produce consistent mixtures, a high-range water-reducing admixture (MasterGlenium 7700 from BASF) was used. The mixtures were then cast into molds and demolded after one day. Two types of molds were used: Cubical molds with overall dimensions of 50 mm × 50 mm × 50 mm and disk molds with dimensions of 50 mm diameter and 25 mm height. After demolding, all samples underwent curing for 28 days in a controlled environment at T = 21 °C and RH > 95%. At the age of 28 days, the cubical samples were cracked using an MTS machine as shown in Fig. 1. The maximum applied load on the specimens was set as the first crack value according to the procedure described by Pang et al.^54^. To monitor the change in crack volume due to autogenous self-healing, a single crack was generated in the disk samples using a screw jack, as shown in Fig. 2.

**Table 1:** Physical and chemical properties of materials used.

Components/property	OPC	CC	MK	BN	FA
Assay percent range (%)	–	> 99	–	–	–
Montmorillonite (%)	–	–	–	85	–
Quartz (%)	–	–	–	5	–
Silicon oxide (SiO_2_) (%)	19.6	–	53.5	–	42.4
Aluminum oxide (Al_2_O_3_) (%)	4.8	–	42.5	–	21.2
Ferric oxide (Fe_2_O_3_) (%)	3.3	–	1.9	–	7.1
Calcium oxide (CaO) (%)	61.50	–	0.20	–	16
Magnesium oxide (MgO) (%)	3	–	–	–	–
Sulfur trioxide (SO_3_) (%)	3.5	–	0.05	–	2.4
Fledspars (%)	–	–	–	5	–
Cristobalite (%)	–			2	–
Loss on ignition (%)	1.90	–	0.5	–	1.60
Insoluble residue (%)	0.44	–	–	–	–
Equivalent alkalis (%)	0.7	–	–	–	–
Tricalcium silicate (C_3_S) (%)	55	–	–	–	–
Dicalcium silicate (C_2_S) (%)	15	–	–	–	–
Tricalcium aluminate (C_3_A) (%)	7	–	–	–	–
Tetracalcium aluminoferrite (C_4_AF) (%)	10	–	–	–	–
Blaine fineness (m^2^/kg)	371	–	–	–	–
Autoclave expansion (%)	0.09	–	–	–	–
Compressive strength 28 days (MPa)	40.9	–	–	–	–
Specific gravity	3.15	2.70	2.60	2.50	2.60

**Table 2:** Physical and chemical properties of sand.

Property	Value
Absorption (%)	1.09
Specific gravity (apparent) (%)	2.72
Specific gravity (dry) (%)	2.65
Specific gravity (SSD) (%)	2.68
Unit weight (kg/m^3^)	1512
Materials finer than 75-μm (sieve # 200) (%)	2.10

**Table 3:** Mixture design of mortars by mass ratio.

Mix	Description	OPC	FA	MK	BN	CC	Sand	Water
1	OPC	100	–	–	–	–	200	35
2	FA20	80	20	–	–	–	200	35
3	MK15	85	–	15	–	–	200	35
4	BN8	92	–	–	8	–	200	35
5	CC8	92	–	–	–	8	200	35

The initial crack width was controlled during the screwing process via a calibration ruler as per the method described by Roig-Flores et al.^55^. For each environmental exposure, three groups of specimens with three different values of crack width were tested. The first group consisted of three specimens with an average crack width in the range of 50–150 μm. For the second and third groups, the average crack widths were 150–300 μm and 300–500 μm, respectively. All cracked specimens were then subjected to diverse environmental conditions including: (i) cyclic temperature (T) from 40 to − 10 °C and cyclic relative humidity (RH) from 90 to 20%; and (ii) water submersion*.*

The crack width change (before and after healing) was inspected via optical microscopy. In order to determine the nature of the healing products formed in the cracks, a scanning electron microscope SU3500 Hitachi High-Tech was employed. Moreover, a computed tomography (CT) system XT 225 ST was used to calculate and quantify changes in the crack total volume imparted by self-healing. The pore size distribution of the cracked samples was analyzed by an AutoPore IV9500 mercury intrusion porosimeter. All specimens were desiccated before testing to achieve constant mass using a glass desiccator.

### Shear wave velocity

**Figure 3:**
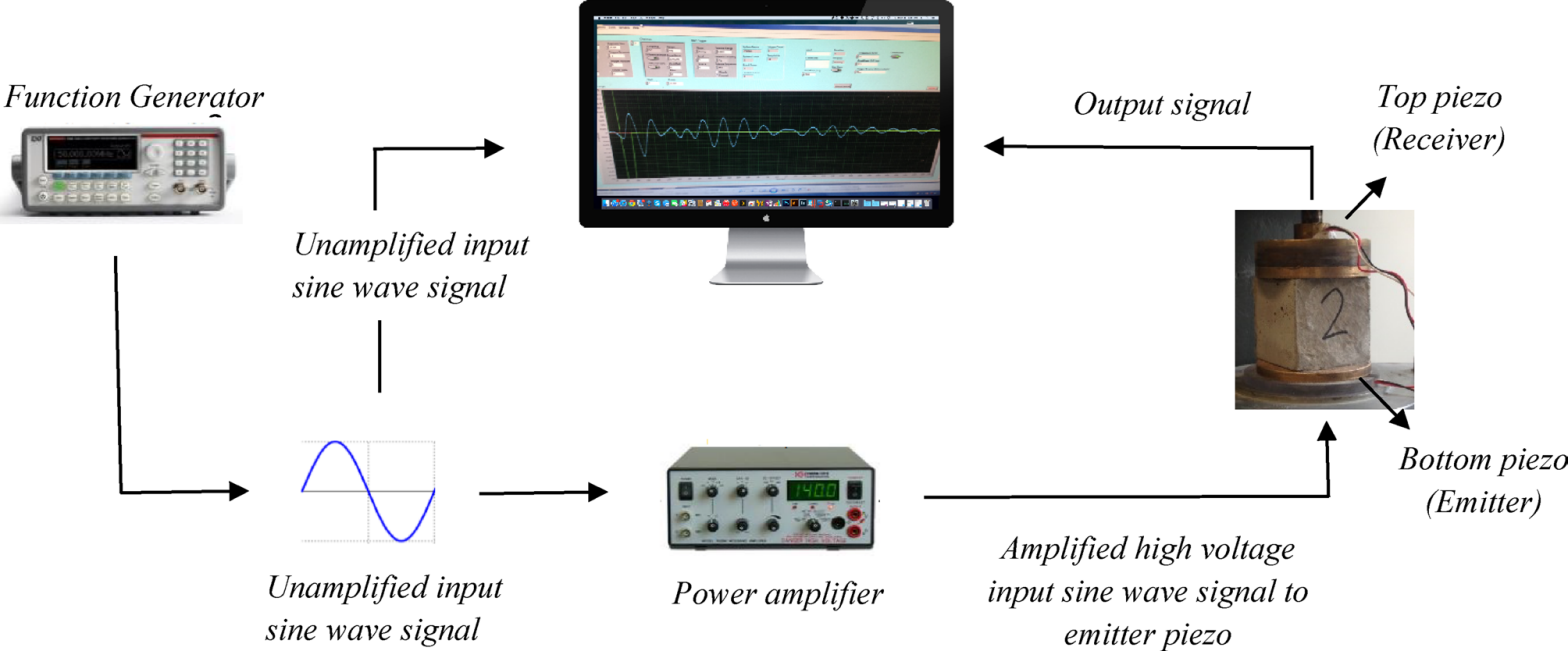
Schematic representation of PRA test set-up.

**Figure 4:**
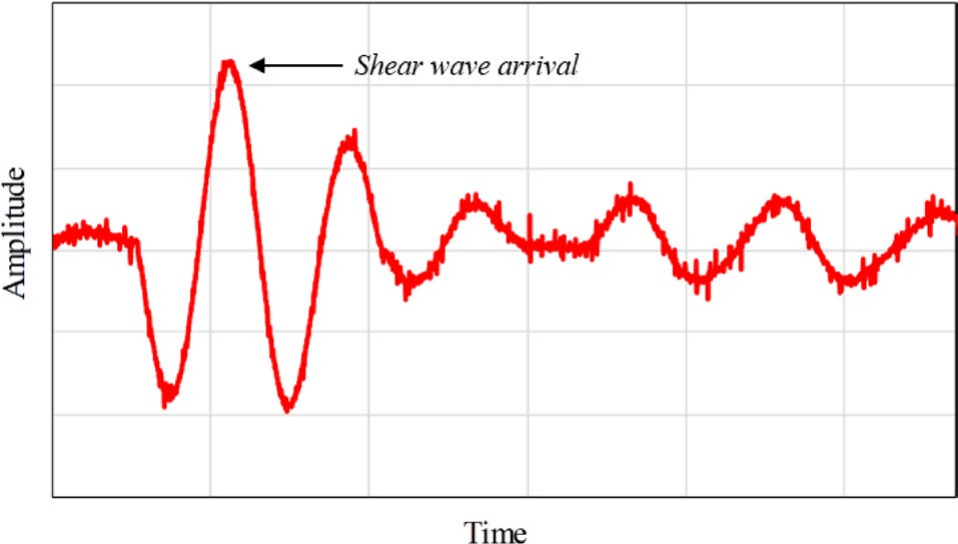
Typical output signal with clear shear wave arrival.

A piezoelectric ring actuator (PRA) technique was used for measuring the shear wave velocity of the tested specimens. This technique was originally developed at Sherbrooke University^56^ and further developed at The University of Western Ontario^57^. Two piezoelectric ring-shaped transducers were mounted on two opposite surfaces of the tested specimens to evaluate the shear wave velocity (Fig. 3). A data acquisition system was used to capture and record the resulting signals. During the test, a sinusoidal wave with high voltage was generated across the specimen with different input excitation frequencies. Figure 4 depicts a typical signal with clear shear wave arrival. The arrival point of the shear wave signal was taken as the first noteworthy signal excursion having positive polarity, as described by Mneina et al.^58^. The variation in SWV before and after cracking and healing was periodically investigated.

## Results and discussion

Considering the ongoing controversy regarding the influence of crack closing induced by autogenous self-healing on mechanical strength recovery of cement-based materials, the authors used several techniques that can explore the multiple mechanisms involved. The used tests compliment each other to provide more robust assessment. Mobilizing multiple test methods can yield synergistic information that better captures the diverse mechanisms governing the relationship between crack closing and mechanical strength recovery.

### Shear wave velocity and strength recovery

**Figure 5:**
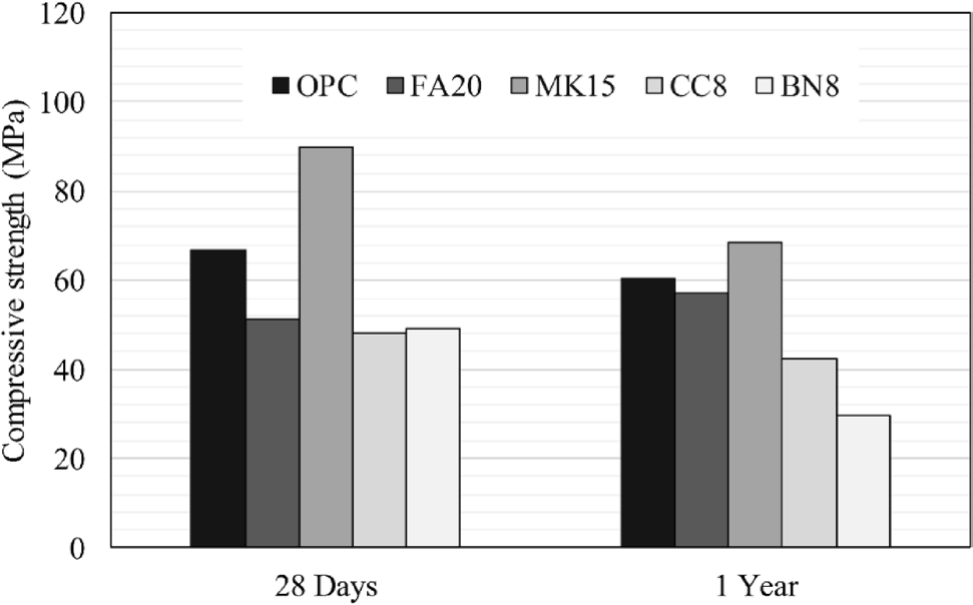
Compressive strength of pre-cracked mortar specimens subsequently submerged in water.

**Figure 6:**
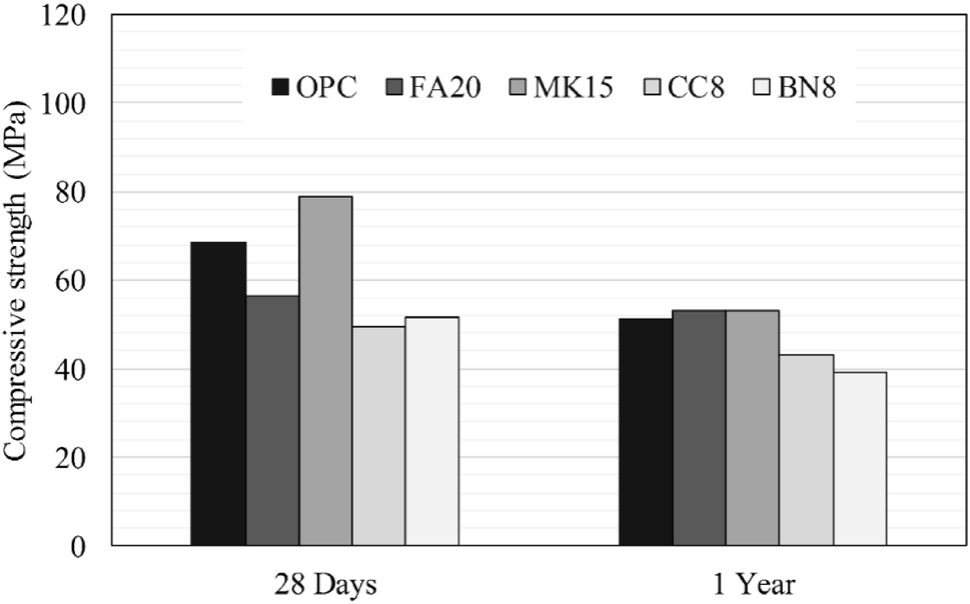
Compressive strength of pre-cracked mortar specimens exposed to cyclic RH and T.

Figures 5 and 6 illustrate the compressive strength of pre-cracked specimens subjected to various environmental exposures. After one year, the cracked and cured specimens were re-cracked to the maximum load. Based on the results, self-strength recovery can hardly be interpreted using a destructive technique such as compression testing. Tables 4 and 5 report the shear wave velocity, SWV evolution of the self-healed specimens exposed to different environments. Several measurements of SWV were taken over 1 year. The test results were normalized with respect to the original value before cracking. For specimens exposed to water submersion, results show that the FA20 specimens achieved significant strength recovery. In contrast, specimens BN8 yielded the least strength recovery. For specimens FA20, strength recovery can be related to the progress of cement hydration reactions and formation of calcium silicate hydrate (CSH) due to pozzolanic reactions. For specimens MK15, although metakaolin can undergo pozzolanic reactions and refine the microstructure of the hydrated cement paste, it reacted quicker than fly ash owing to its smaller particle size and higher surface area. Conversely, bentonite in BN8 specimens contains montmorillonite, which is a crystalline material that has less contribution to C–S–H formation, resulting in less strength recovery. Similarly, CC8 specimens incorporating calcium carbonate exhibited low strength recovery. According to Kenai et al.^59^ fine calcium carbonate powder may reduce the long-term compressive strength due to the cement hydration dilution effect induced by calcium carbonate powder, as observed by^60^.

**Table 4:** Shear wave velocity evolution of mortar specimens submerged in water.

	Normalized SWV				
	Uncracked	Cracked	3 months	6 months	1 year
OPC	1	0.55	0.64	0.77	0.79
FA 20	1	0.59	0.68	0.78	1.12
MK 15	1	0.51	0.64	0.74	0.81
BN 8	1	0.56	0.71	0.72	0.75
CC 8	1	0.53	0.70	0.72	0.77

**Table 5:** Shear wave velocity evolution of mortar specimens exposed to cyclic T and RH.

	Normalized SWV				
	Uncracked	Cracked	3 months	6 months	1 year
OPC	1	0.58	0.61	0.64	0.65
F20	1	0.52	0.62	0.66	0.69
MK15	1	0.53	0.59	0.65	0.66
BN8	1	0.58	0.59	0.64	0.65
CC8	1	0.54	0.57	0.59	0.61

In the case of specimens subjected to cyclic RH and T (Table 5), the self-strength recovery was considerably lower than that of specimens exposed to continuous water submersion. This can be ascribed to the fact that further hydration and pozzolanic reactions in specimens exposed to cyclic RH and T were significantly diminished. For example, previous study by Özbay et al.^34^ showed that mechanical strength recovery of pre-cracked ECC specimens continuously cured in ambient air was significantly reduced in comparison to that of identical samples cured in water. Similar results were reported by Sisomphon et al.^61^ when they investigated strain hardening cementitious composites incorporating calcium-sulfo-aluminate crystalline and expansive additives. Their results indicated that all specimens exposed to ambient air had minimal mechanical strength recovery in compassion to specimens exposed to damp environments. This indicates that self-strength recovery of a cement-based material predominantly depends on the moisture conditions of the surrounding environment. Therefore, further hydration of cementitious materials, which requires the presence of a damp environment, seems to be the main mechanism for mechanical strength recovery in cement-based materials.

### Crack self-healing and strength recovery

**Figure 7:**
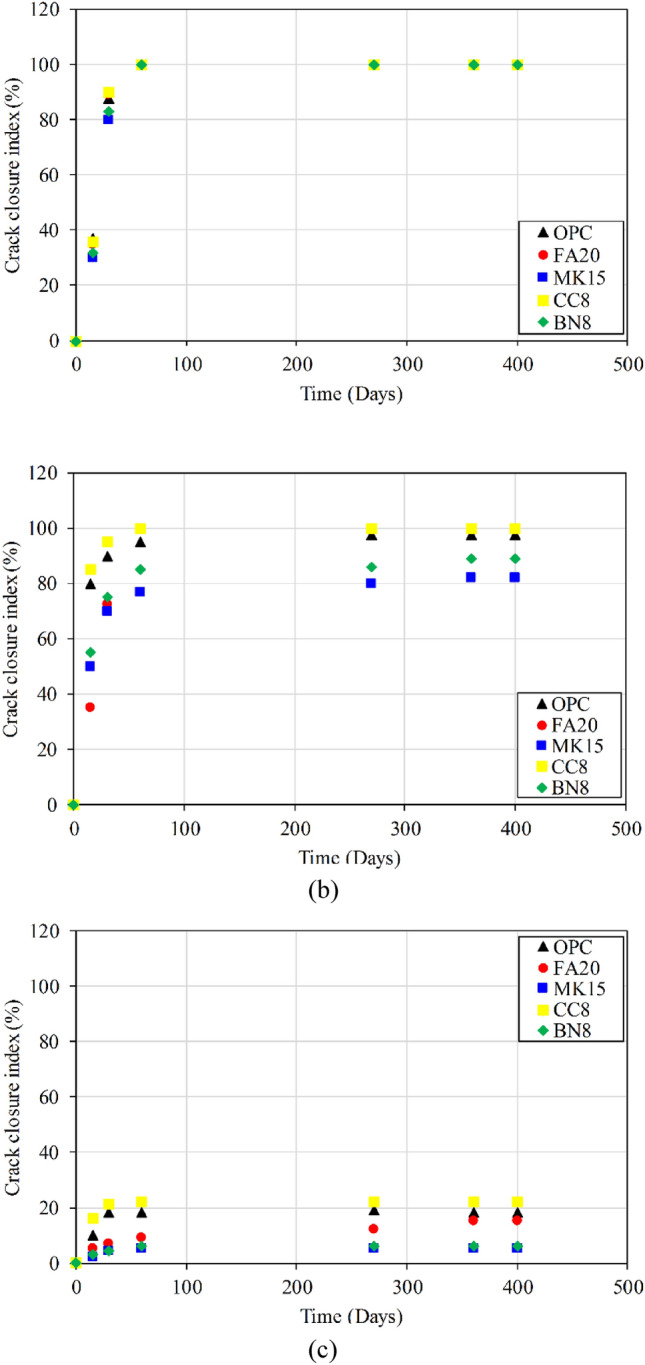
Illustration of change in surface crack width incurred by water submerged mortar specimens (initial crack width: (**a**) 50–150 µm; (**b**) 150–300 µm; and (**c**) 300–500 µm.

**Figure 8:**
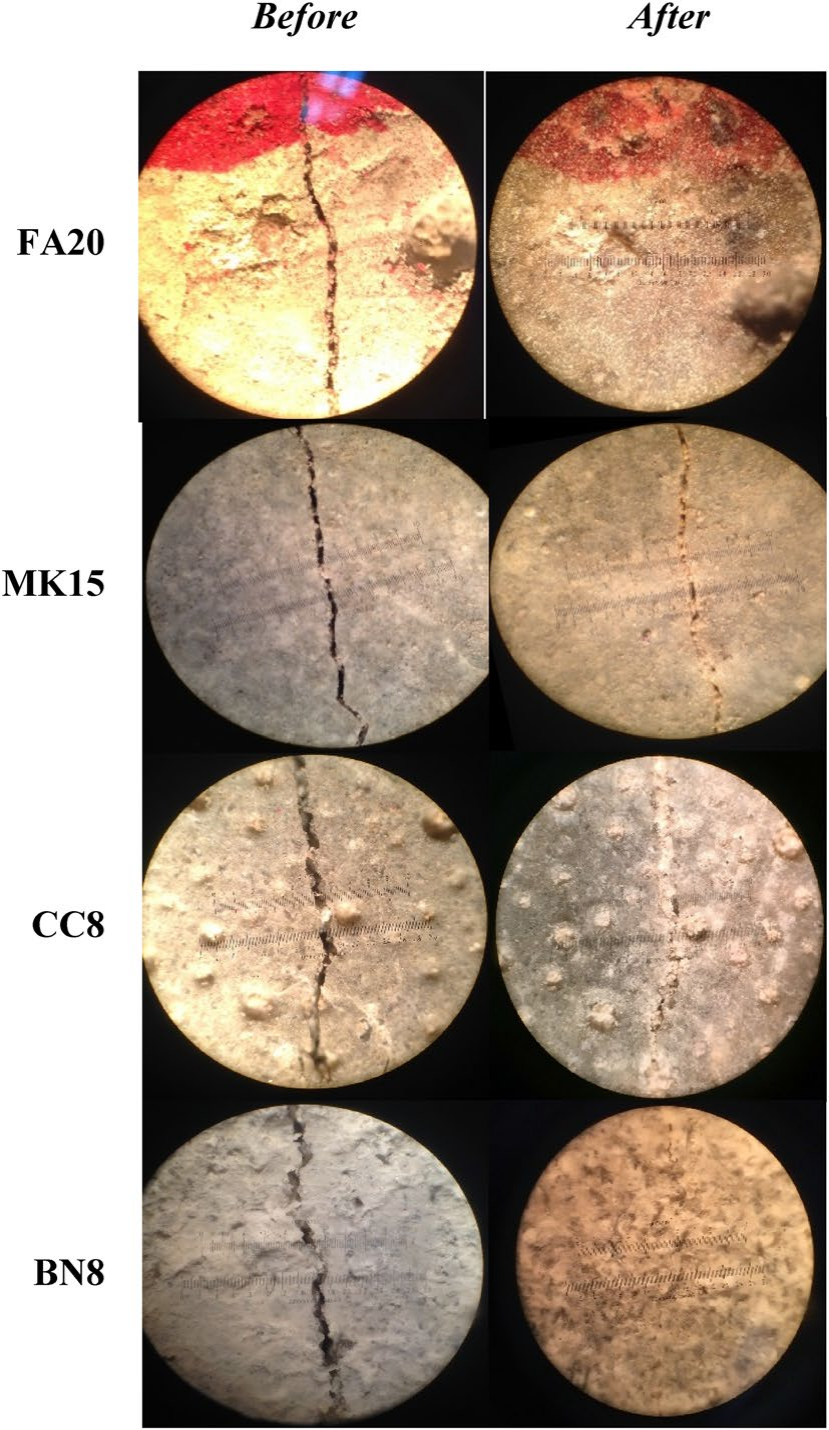
Initial cracked surfaces and status after water submersion exhibiting self-healing.

Figure 7 depicts the crack sealing index of specimens after 400 of water immersion. Results indicate that samples cured in water achieved self-healing (Fig. 8). On the other hand, the crack width in all specimens subjected to cyclic RH and T remained relatively unchanged. Several studies have shown that cement-based materials exhibit self-healing primarily when the curing environment is conducive to contact with liquid water^19,23,27,33,34,38,55,61–65^. For example, Sahmaran et al.^23^ investigated the self-healing of surface cracks in cementitious composites exposed to continuous wet, continuous air, and freeze–thaw cycles. The rapid chloride permeability test was used to evaluate the rate of self healing. Their results showed that the continuous wet condition had more pronounced effect on chloride ion permeability, indicating the importance of the presence of water for self-healing. Luo et al.^64^ showed that water curing was necessary for bacteria-based self-healing concrete. Similarly, Roig-Flores et al.^55^ reported that the presence of water was essential for the self-healing process.

For crack self-healing to occur, it seems that both H_2_O and CO_2_ need to be present inside the crack. As Ca^2+^ ions start to get released into the pore solution within the crack, calcium carbonate (CaCO_3_) will precipitate and fill the crack, leading to crack self-healing, as per the reaction:

$${\text{Ca}}\left( {{\text{OH}}} \right)_{2} + {\text{CO}}_{2} \to {\text{CaCO}}_{3} + {\text{H}}_{2} {\text{O}}.$$

This process can occur any time during the concrete's lifespan as long as H_2_O and CO_2_ are present inside the crack. On the other hand, the hydration process of concrete has a time limit, which depends on the concrete constituents and curing procedure. Therefore, autogenous crack self-healing could progress even after the hydration process has entirely been completed.

**Figure 9:**
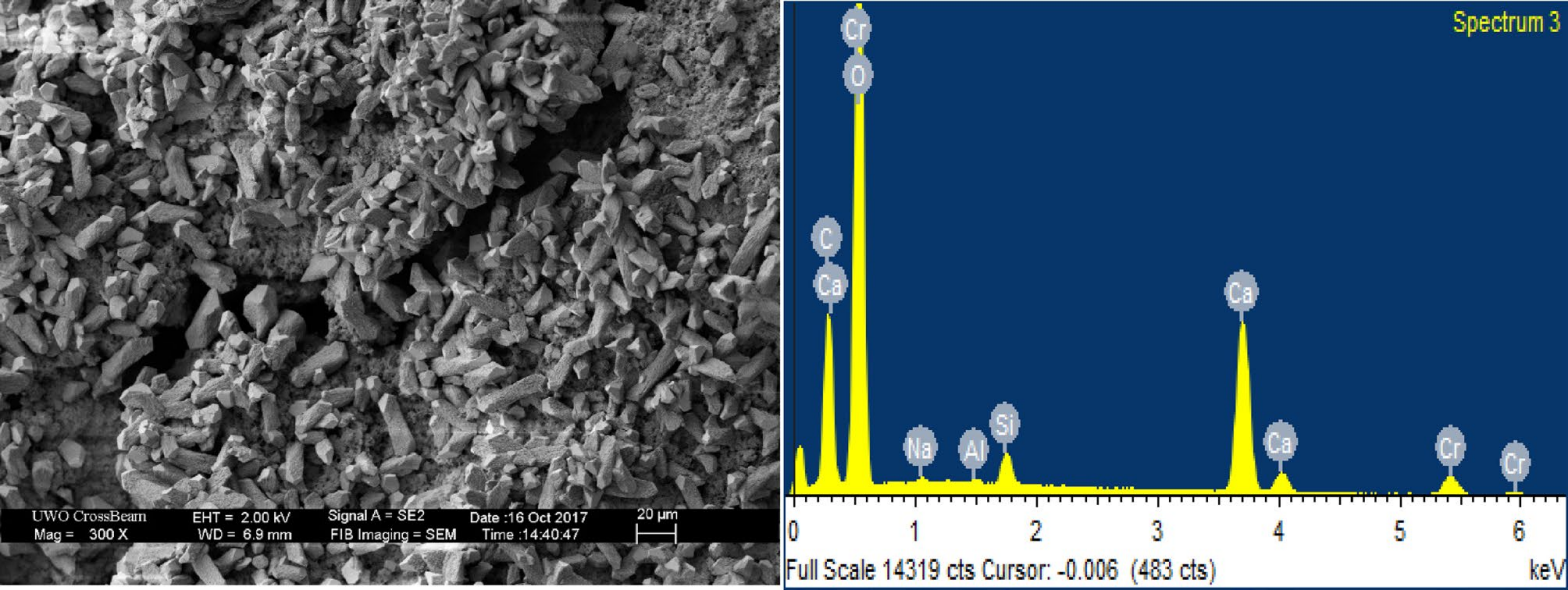
SEM micrograph with EDX pattern of products in self-healed cracks of CC8 specimens.

**Figure 10:**
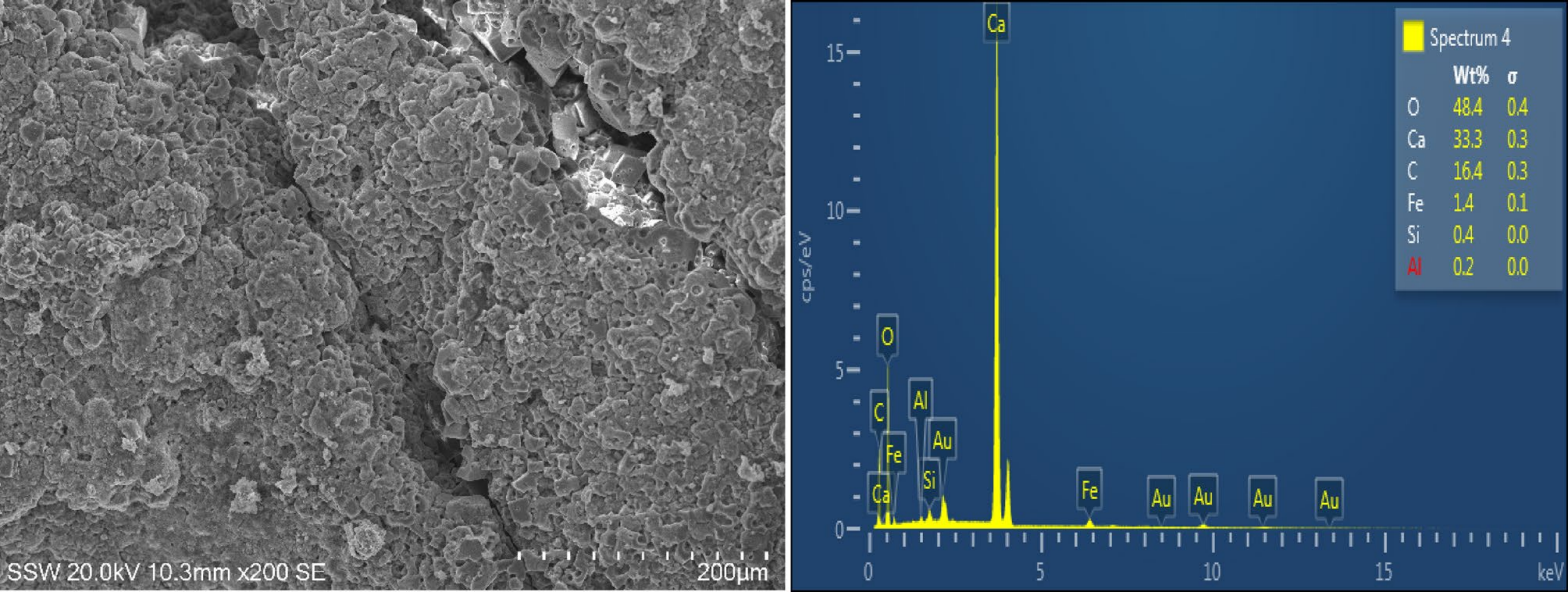
SEM images with energy disperse X-ray pattern of compounds in self-healed cracks for FA20 specimens.

In the present study, mortar specimens incorporating fine calcium carbonate powder showed higher tendency of surface crack healing than the other specimens. SEM and EDX results for the healing products formed on the surface cracks of CC8 specimens indicate the formation of CaCO_3_ (Fig. 9). However, in terms of strength recovery, CC8 specimens did not achieve appreciable improvement in compressive strength in comparison with results of FA20 specimens. For the FA20 specimens, SEM and EDX results also showed that the healing compound which emerged at the surface of cracks was primarily CaCO_3_ (Fig. 10).

**Figure 11:**
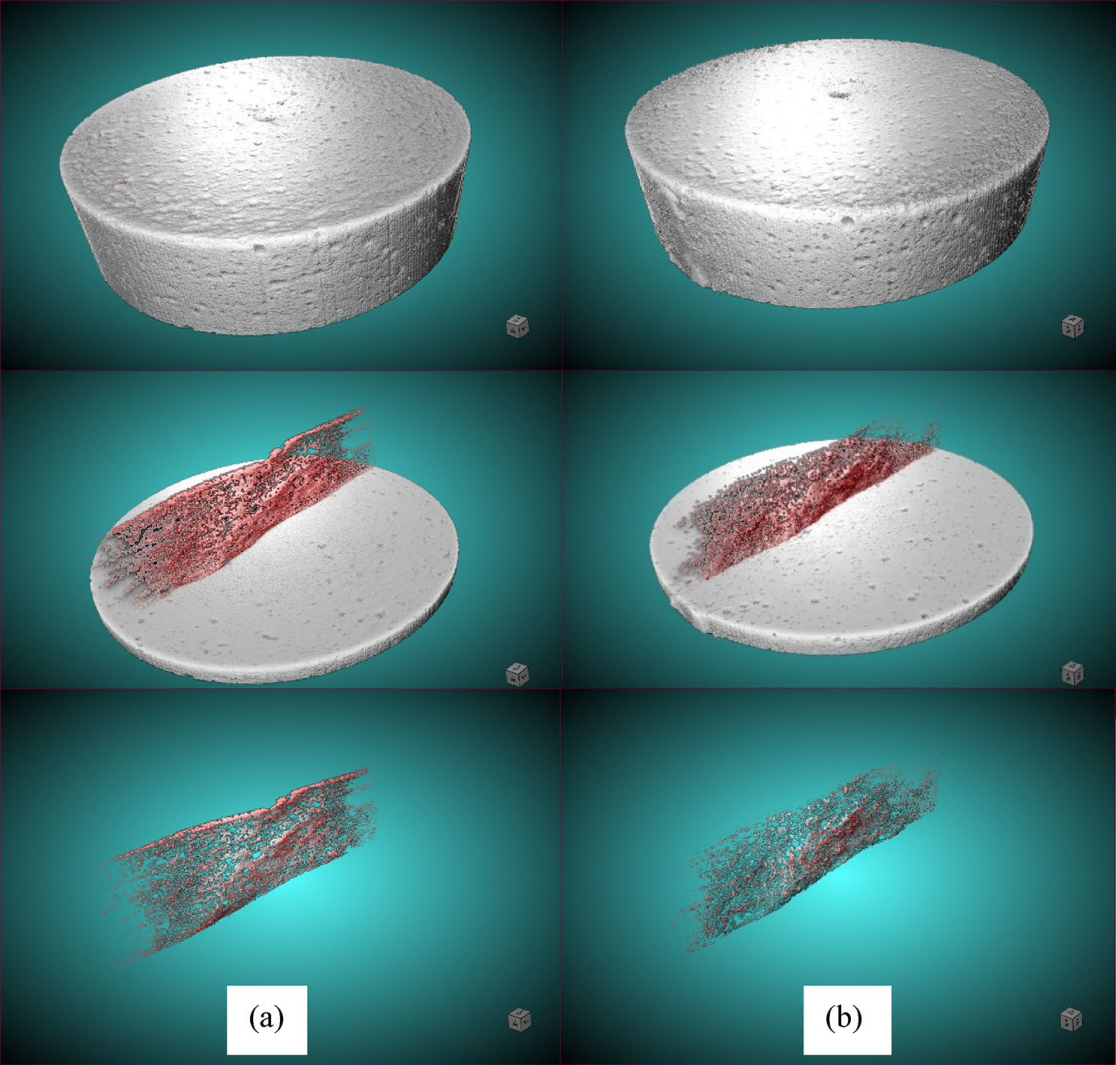
X-ray µCT scan images for FA20 specimen submerged in water depicting: (**a**) before process of self-healing, and (**b**) after process of self-healing.

Figure 11 shows X-ray µCT scan images for FA20 specimens (before and after self-healing). To calculate the self-healing efficiency, the change in size of the entire crack volume owing to self-healing was quantified using a Nikon XT-H-225-ST μCT scanner housed at Sustainable Archaeology, Western University. On the day of cracking, specimens with crack width in the range of 150–300 μm were scanned. However, only specimens that exhibited surface crack self-healing were scanned for a second time. The X-ray projections were harvested to reconstruct the exterior and interior of the sample using the Nikon's CT-Pro (v 4.4.3) reconstruction software. Parameters of the scan were defined as follows: voltage = 225 kVp; current = 60 μA (power of 13.5 W). A 1-mm Cu filter was used to filter the X-ray beam with 3141 projections gathered during a 53 min scan. Two datasets (at the day of cracking and after self-healing) were input into a 3D rendering software (Dragonfly 3.5) developed by Object Research Systems (ORS). Dragonfly 3.5 is an advanced image processing, segmentation, and quantification analysis tool. It can import data of different types, sizes, and scales to measure and quantify objects within multi-region of interests such as cracks.

Results from Dragonfly 3.5 illustrate that the crack self-healing efficiency was 26.95%, 32.26%, 27.27%, 25.6%, and 24.1% for specimens FA20, CC8, OPC, BN8, and MK15, respectively. Hence, it can be posited that specimens FA20 achieved slightly less crack healing than that of CC8 and OPC specimens, and a little more crack healing than that of MK15 and BN8. However, in terms of strength recovery, FA20 achieved significant strength recovery in comparison to the other specimens (e.g. FA20 > MK15 > OPC > CC8 > BN8). Apparently, specimens incorporating silica-based additions (pozzolanic materials) such as fly ash achieved higher strength recovery than the other specimens incorporating swelling compounds and carbonating minerals. This indicates that the strength recovery achieved by FA20 specimens can be related to the progress of the hydration process and further formation of CSH due to pozzolanic reactions.

This is further supported by work of Şahmaran et al.^37^ who investigated the mechanical strength recovery of ECC specimens, which either incorporated class F fly ash or slag, using resonant frequency (RF) tests. Their results showed that ECC specimens incorporating class F fly ash attained higher recovery of RF than that of ECC specimens incorporating slag. However, in terms of crack closing due to self-healing, the same ECC specimens incorporating slag yielded better performance than that of the ECC specimens containing Class F fly ash. According to Şahmaran et al.^37^, the ECC specimens incorporating Class F fly ash achieved better RF recovery due to further hydration and pozzolanic reactions owing to the abundant un-hydrated Class F fly ash particles that persisted even at later ages. Conversely, ECC specimens made with slag contained a higher amount of calcium oxide, which led to better crack closing ability than ECC specimens made with Class F fly ash, as reported elsewhere by Sahmaran et al.^23^. This indicates that surface crack self-healing may have little or negligible contribution to mechanical strength recovery of cement-based materials.

A previous study by Yang et al.^39^ investigated the autogenous healing of ECC subjected to wetting and drying cycles. When specimens were exposed to tensile loading after experiencing self-healing, it was observed that most newly formed cracks tended to follow the previous crack lines as reported elsewhere by Zhu et al.^33^. According to Yang et al.^39^, the healing products in cracks, mainly CaCO_3_ crystals, had a weak bond and were relatively weaker than the primary hydrated cementitious matrix pozzolanic reactions.

In the present study, images from optical microscopy and X-ray computed tomography showed that complete self-healing was mostly concentrated at the surface of specimens (Figs. 8 and 11). This indicates that autogenous crack self-healing in cement-based materials has minimal or negligible effect on mechanical strength recovery. Therefore, it can be hypothesized that strength recovery of cementitious materials reported in diverse studies could rather be attributed to ongoing cement hydration and/or pozzolanic reactions in the un-cracked portion of the matrix rather than to effective strength recovery via healing of cracks. The images in Fig. 11 were generated using the Dragonfly software, Version 3.5 for [Windows], Object Research Systems (ORS) Inc., Montreal, Canada, 2018. The software is available at http://www.theobjects.com/dragonfly.

### Microstructural densification and strength recovery

**Figure 12:**
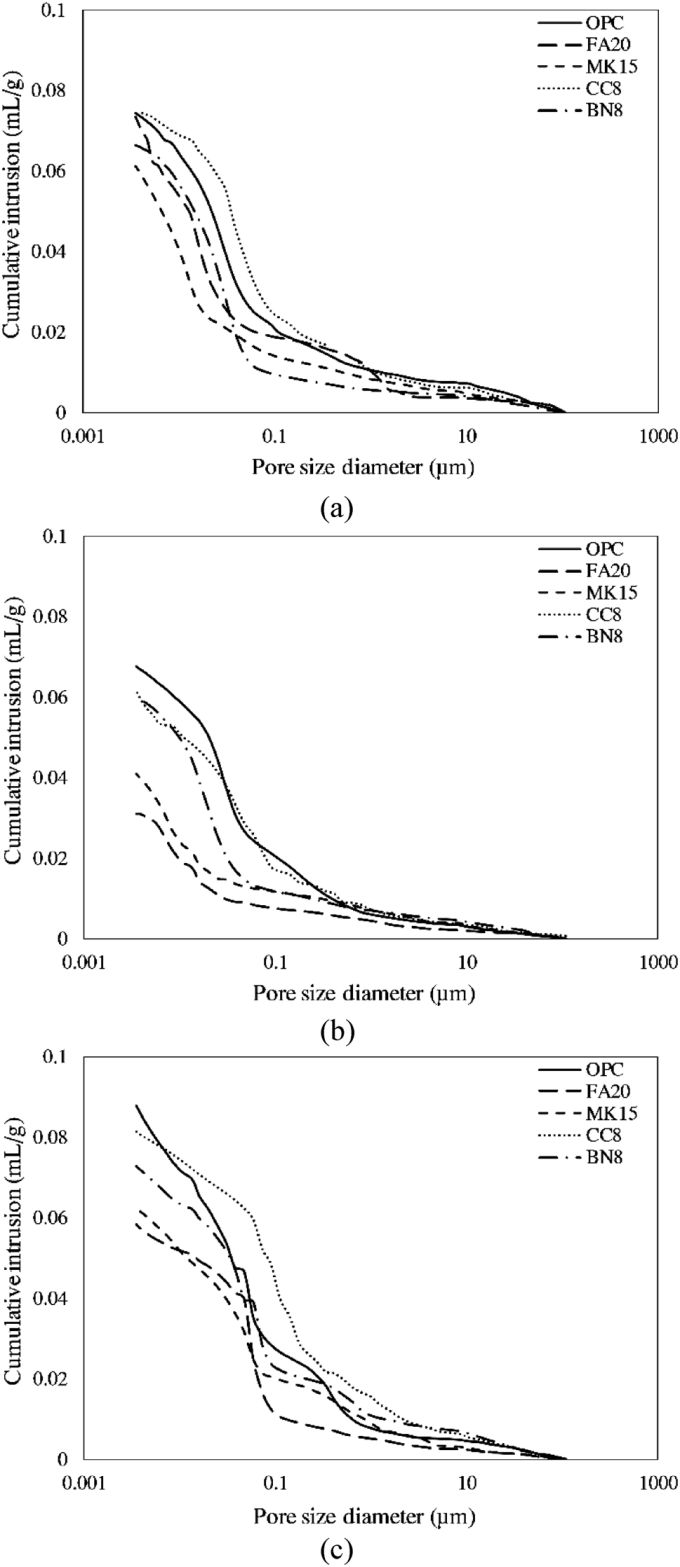
Illustration of cumulative MIP intruded pore volume versus pore diameter for cracked specimens: (**a**) at 28-day, (**b**) after 1-year of water submersion, and (**c**) after 1-year of cyclic T and RH.

Figure 12 shows MIP results for the tested specimens subjected to various environments. Fragments from the uncracked portion of the tested specimens (before and after self-healing) were studied using a Micrometrics AutoPore IV 9500 Series porosimeter. Results indicate that water submerged specimens (particularly FA20) achieved significant reduction in porosity compared with that of specimens subjected to cyclic RH and T. In addition, although FA20 specimens attained less crack healing than that of CC8 and OPC after 1 year of water submersion, they achieved the highest reduction in porosity. Reduction in porosity for specimens containing fly ash resulted primarily from the progress of pozzolanic reactions, which promoted microstructural densification and pore refinement, resulting in mechanical strength enhancement.

This indicates that the strength recovery of FA20 specimens was essentially produced by delayed hydration and pozzolanic reactions, rather than by precipitation and crystallisation of CaCO_3_ in the healed cracks. In a previous study, Ferrara et al.^63^ investigated mechanical properties recovery of concrete incorporating crystalline admixtures. They found that the mechanical recovery was attributed to further hydration reactions rather than to conversion of Ca(OH)_2_ into CaCO_3_. Hilloulin et al.^66^ studied the mechanical strength recovery in mortar specimens made with limestone portland cement cracked at different ages. Their results showed that specimens cracked at the age of 24 h achieved higher strength recovery in comparison to that of similar specimens cracked at the age of 72 h or later. According to Hilloulin et al.^66^, the lack of remaining un-hydrated products in specimens cracked at the age of 72 h or later was the main reason for reducing strength recovery in these specimens. Sisomphon et al.^61^ explored the self-healing potential of strain-hardening cementitious composites. Their results showed that although the formation of CaCO_3_ is desirable with regards to water tightness, it may decrease the recovery of mechanical properties. According to Qian et al.^38^, healing products formed in cracks, including CaCO_3_ and Ca(OH)_2_, are typically weaker than CSH gel. Even if the main healing product in cracks was CSH formed from cement particle rehydration, the bond strength of the crack surface to the newly formed CSH would be weaker in comparison with the tensile strength of the uncracked matrix.

In the current study, although FA20 specimens had slightly less crack volume change due to self-healing compared with that of the CC8 and OPC specimens, their strength recovery was significantly higher. This could be attributed to the progress of hydration and pozzolanic reactions, which is related to strength enhancement of the uncracked matrix, rather than the healing of cracks. Hence, it appears at this stage that surface crack healing via deposition of calcium carbonate and hydrated lime may play a significant role in regaining durability features though reducing mass transport through the surface via enhancing gas and fluid-tightness and restricting the intrusion of deleterious ions. However, its contribution to mechanical strength recovery appears negligible based on the findings of the present study.

## Conclusions

In this study, the influence of autogenous crack self-healing in cement mortar on its mechanical strength recovery under various exposure environments was investigated using a wide array of multi-scale experimental techniques. Moreover, the effects of microstructural changes in mortar induced via additives such as swelling compounds, silica-based additions, and carbonating minerals on its mechanical strength recovery under diverse environmental exposures was studied. Based on the experimental findings and insights, the following concluding remarks can be drawn:

1. The exposure condition plays a crucial role in the mechanical strength recovery process. The water submersion condition achieved the best strength recovery, particularly for specimens incorporating fly ash.
2. Specimens incorporating limestone microfiller achieved the best crack healing ability in terms of crack filling and closing.
3. In terms of strength recovery, specimens incorporating fly ash attained the best performance.
4. Based on shear wave velocity measurements, the ability of specimens to achieve mechanical strength recovery was ranked in decreasing order as FA20 > MK15 > OPC > CC8 > BN8.
5. Based on high-resolution X-ray computed tomography and 3D image analysis, the crack healing effectiveness of tested specimens was in the decreasing order of CC8 > OPC > FA20 > BN8 > MK15.
6. Experimental results indicate that autogenous crack self-healing in cement-based materials has a negligible effect on their ability to achieve mechanical strength recovery. It appears that latent cementitious hydration and pozzolanic reactions would be the primary cause for cement-based materials to exhibit mechanical strength recovery.
7. Mechanical strength recovery seems to be limited to the duration of the hydration and pozzolanic reactions process of cement-based materials, while autogenous crack self-healing could progress even after the hydration process has entirely been completed.
8. While autogenous self-healing in favorable moist environments could play a significant role in sealing the surface of cement based materials against the ingress of hostile media, and thus may regain some durability features, re-establishing mechanical strength after cracking would require other self-healing mechanisms such as bacteria-, vascular- and encapsulation-based self-healing mechanisms.
9. The results of this study apply to portland cement-based materials, which represent by far the largest construction material used on earth, and the most consumed commodity after water. However, the results need to be validated for emerging binders such as geopolymers, alkali-active systems and other classes of non-portland based cements.
